# Supplementary material for: Comparative Study on Different Remediation Strategies Applied in Petroleum-Contaminated Soils
Source: Int J Environ Res Public Health. 2020 Mar 2;17(5):1606. doi: 10.3390/ijerph17051606 (PMC7084466; doi:10.3390/ijerph17051606)
Supplement: Supplementary file 1 [file ijerph-17-01606-s001.pdf]

Table S1. Contents of ten PHs in the initial added stage (mg/kg).

Petroleum Hydrocarbon	Tridecane	Tetradecane	Pentadecane	Hexadecane	Heptadecane	Octadecane	Eicosane	Heneicosane	Naphthalene	Phenanthrene
Contents	1015.18 ± 54.48	1015.47 ± 87.12	1029.48 ± 42.85	1058.41 ± 78.26	1045.74 ± 29.15	1035.18 ± 98.26	1048.18 ± 105.25	1036.24 ±3 9.15	1019.36 ± 24.49	1014.33 ± 83.47

Table S2. Phylum of bacteria community in the remediation process.

**Order**	Phyla	**Order**	Phyla	**Order**	Phyla
1	*Acidobacteria*	19	*Ignavibacteriae*	37	*WS6*
2	*Actinobacteria*	20	*Latescibacteria*	-	-
3	*Armatimonadetes*	21	*Lentisphaerae*	-	-
4	*BRC1*	22	*Microgenomates*	-	-
5	*Bacteroidetes*	23	*Nitrospirae*	-	-
6	*Chlamydiae*	24	*Parcubacteria*	-	-
7	*Chlorobi*	25	*Planctomycetes*	-	-
8	*Chloroflexi*	26	*Proteobacteria*	-	-
9	*Cyanobacteria*	27	*RBG-1_[Zixibacteria]*	-	-
10	*Deferribacteres*	28	*Saccharibacteria*	-	-
11	*Deinococcus-Thermus*	29	*Spirochaetae*	-	-
12	*Elusimicrobia*	30	*Synergistetes*	-	-
13	*FBP*	31	*TM6_[Dependentiae]*	-	-
14	*Fibrobacteres*	32	*Tectomicrobia*	-	-
15	*Firmicutes*	33	*Tenericutes*	-	-
16	*Fusobacteria*	34	*Unassigned*	-	-
17	*GAL15*	35	*Unclassified*	-	-
18	*Gemmatimonadetes*	36	*Verrucomicrobia*	-	-

Table S3. Soil enzyme activities in different remediation strategies (after 126 days).

**Enzyme**	**S0**	**S1**	**S2**	**S3**	**S4**
CAT	1.76 ± 0.09a	1.82 ± 0.15a	2.34 ± 0.21b	2.64 ± 0.14b	3.79 ± 0.25c
LPS	8.57 ± 0.52a	10.39 ± 0.78b	14.71 ± 1.15c	11.29 ± 0.78b	22.74 ± 1.36d
DDA	0.64 ± 0.04a	0.67 ± 0.04a	1.62 ± 0.12b	1.91 ± 0.12c	2.47 ± 0.15d
PPS	151.71 ± 11.66a	162.32 ± 12.36a	248.23 ± 20.26b	173.29 ± 11.36a	361.75 ± 17.69c
FDA	172.95 ± 13.66a	184.95 ± 9.36a	193.26 ± 10.26a	277.25 ± 11.36b	441.73 ± 17.69c

Lower-case letters in the same line indicate significant differences at $p < 0.05$, $n = 3$.

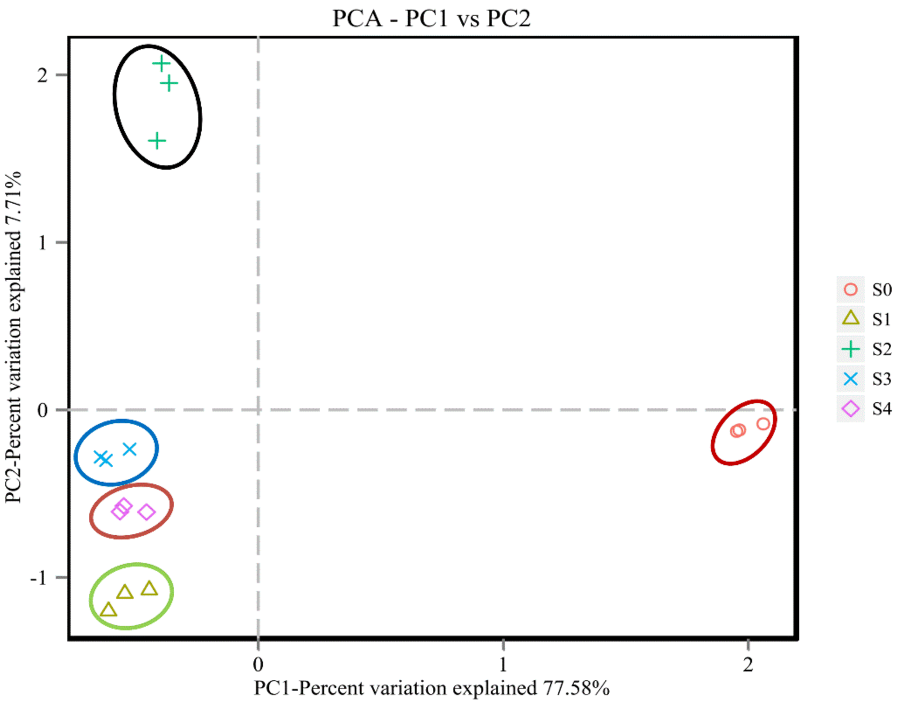

Figure S1. Beta-diversity of bacteria community in different remediation strategies.

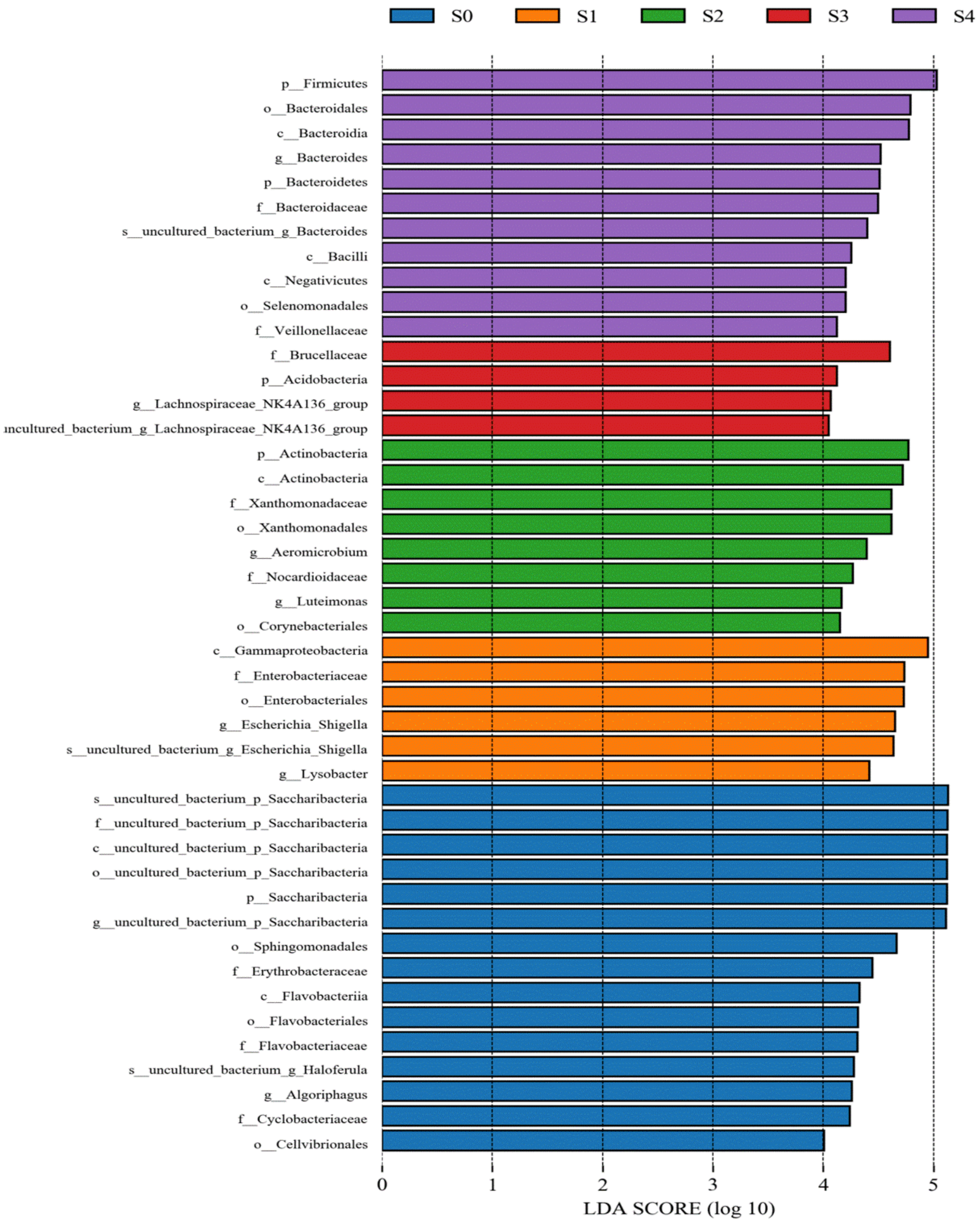

Figure S2. Linear discriminant analysis (LDA) values in different remediation strategies. The x-axis values represent LDA values.

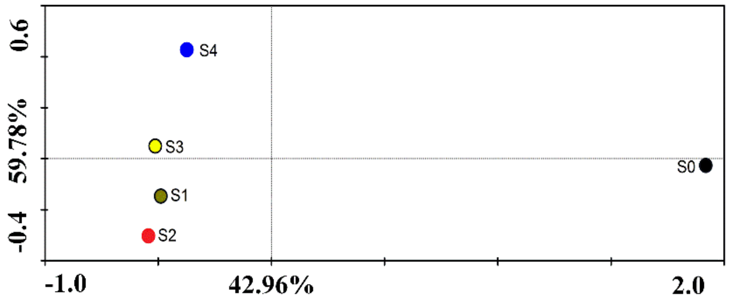

Figure S3. Result of PCA in different remediation strategies

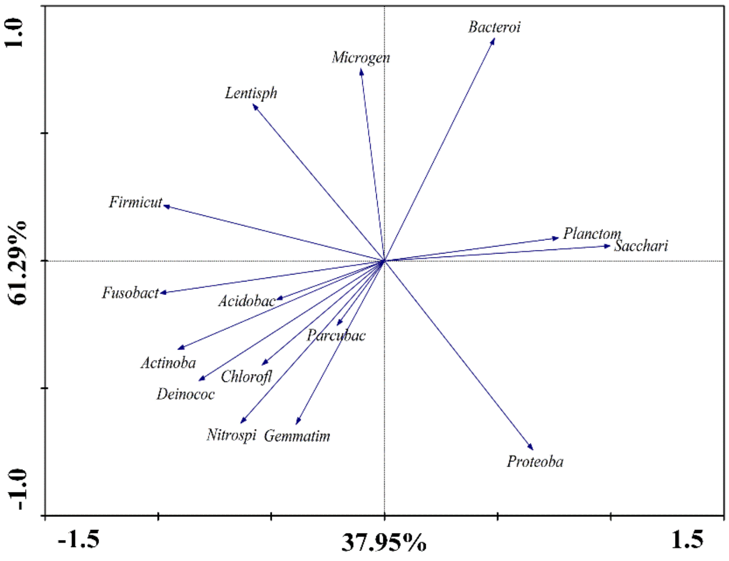

Figure S4. Relationship among bacteria in the community.

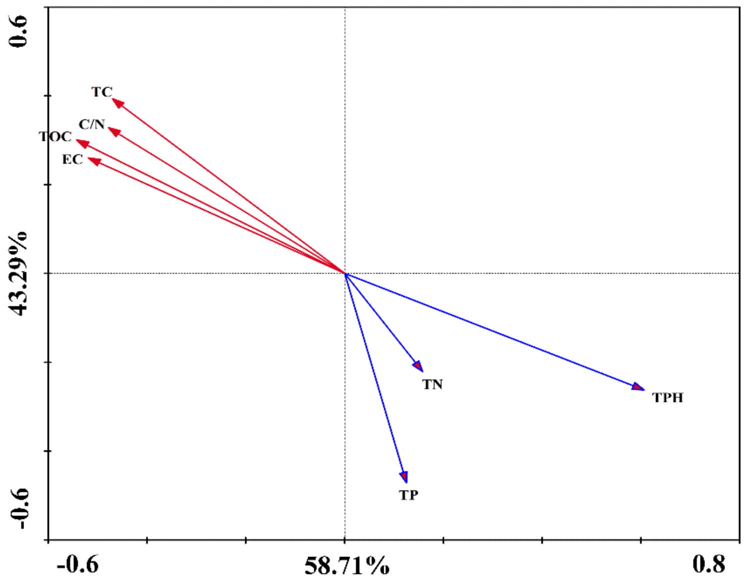

Figure S5. Relationship among physicochemical soil characteristics.

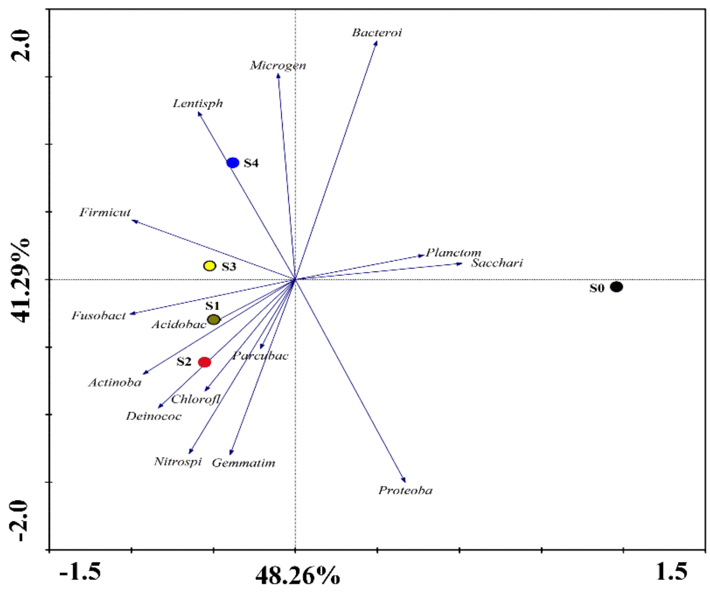

Figure S6. Relationship between the bacterial community and remediation strategies.

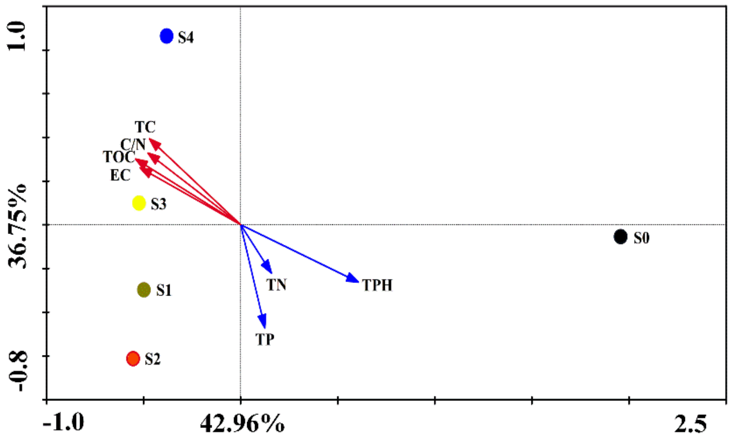

Figure S7. Relationship between physicochemical soil characteristics and remediation strategies.
